# Abortion and conscientious objection: rethinking conflicting rights in the Mexican context

**DOI:** 10.1080/11287462.2017.1411224

**Published:** 2017-12-08

**Authors:** Gustavo Ortiz-Millán

**Affiliations:** ^a^ Instituto de Investigaciones Filosóficas, Universidad Nacional Autónoma de México, Mexico City, Mexico

**Keywords:** Abortion, conscientious objection, reproductive rights, restrictions to conscientious objection, conscience absolutism, Mexico City

## Abstract

Since 2007, when Mexico City decriminalized abortion during the first trimester, a debate has been taking place regarding abortion and the right to conscientious objection (CO). Many people argue that, since the provision of abortions (or “legal terminations of pregnancy” as they are called under Mexico City’s law) is now a statutory duty of healthcare personnel there can be no place for “conscientious objection.” Others claim that, even if such an objection were to be allowed, it should not be seen as a right, since talk about a right to CO may lead to a slippery slope where we may end up recognizing a right to disobey the law. In this paper, I argue that there *is* a right to CO and that this may be justified through the notions of autonomy and integrity, which a liberal democracy should respect. However, it cannot be an absolute right, and in the case of abortion, it conflicts with women’s reproductive rights. Therefore, CO should be carefully regulated so that it does not obstruct the exercise of women’s reproductive rights. Regulation should address questions about who is entitled to object, how such objection should take place, and what can legitimately be objected to.

## Conscientious objection as a form of boycott

Following the approval in April 2007 of the law reform decriminalizing first-trimester abortion in Mexico City, this procedure immediately began to be put into effect in public hospitals run by the city’s Department of Health (DoH). However, many of the physicians and healthcare personnel working in those hospitals (about 88.5%, i.e. 256 out of 289 people in 13 hospitals) declared themselves conscientious objectors and refused to perform abortions. Anesthesiologists, nurses, admission staff, and patient transportation staff, among some others, refused to provide any kind of service to women seeking abortions. Radiologists would not perform ultrasounds, forcing women to provide their own studies. After few months, the DoH stated that only OB/GYNs and general surgeons actively performing the procedure could invoke conscientious objection (CO) (Contreras, Van Dijk, Sanchez, & Sanhueza-Smith, 2011). Later on, a modification to Mexico City’s General Health Law enacted in September 2009 established that

The physician who is responsible for practicing legal termination of pregnancy [ILE or *interrupción legal del embarazo* in Spanish] and whose beliefs, religion or personal convictions are contrary to this procedure, may be a conscientious objector and therefore excused from intervening in the termination of pregnancy, having the obligation to refer the woman to a non-objector physician. When the termination of pregnancy is urgent to safeguard the health or life of the woman, conscientious objection cannot be invoked. It will be the obligation of public health institutions to ensure the timely provision of services and the permanent availability of non-conscientious personnel in the field. (Chapter IX, article 59)^1^

Although in the beginning there was a risk of being unable to implement the ILE program in the city because of the large number of objectors, very soon the service was guaranteed because the DoH made sure that there were enough active physicians to perform the procedures. From April 2007 to November 2017, there have been 186,104 procedures performed in 13 clinics, five of them specialized in maternal health services. Today, there are 19 physicians (out of 270), 20 nurses and 15 social workers, who offer the procedure in public medical facilities; they see some 40–50 women every day (76% of the procedures are medical abortions, 22% vacuum aspiration abortions, and only 2% surgical abortions).^2^

Throughout the country, only Mexico City allows elective or voluntary abortion; each state has its own criminal code and its own regulations regarding health care. Most states allow abortions on three grounds: rape, risk of maternal death and in cases of genetic or congenital anomalies.^3^ However, only six out of 31 states’ laws refer to the CO of healthcare personnel (Aguascalientes, Colima, Jalisco, Mexico City, Querétaro and Tlaxcala); some of these allow for CO without restrictions and do not guarantee that hospitals have non-objecting personnel (GIRE, 2015).

From the moment when the Mexico City reform was implemented, a debate about CO in medicine started and there were questions regarding whether it was justified. While conservatives in Mexico City lost the battle over the decriminalization of first-trimester abortion in both the Legislature and the Supreme Court,^4^ they still hoped to win a battle through CO: even if the right to abortion was guaranteed by law, they wanted to ensure that, in practice, there would be neither doctors nor healthcare personnel willing to guarantee this right. So the conservative National Action Party’s (PAN) leaders launched a campaign calling on staff to object in conscience. CO was used to conceal a boycott of the law for religious reasons, a purposeful strategy to hinder the exercise of women’s reproductive rights.^5^ From 2007 on, conservative politicians have presented several bills in Congress to amend the federal General Health Law – to which all the states’ laws are subordinated – on the issue of CO (the most recent one was approved by the lower house of Congress on October 2017, and it is yet to be discussed by the Senate). These bills were not only a reaction to the issue of abortion, aimed at protecting those opposed to it; they were also intended to cover healthcare personnel who refused to participate in other procedures such as assisted reproduction (particularly for same-sex couples or single women), contraceptive sterilization, prenatal diagnosis for eugenic purposes, treatment of sexually active gay patients, active euthanasia, and assisted suicide, among others. In their conception of CO, once a healthcare provider objects in conscience, this person is to be regarded as exempted from performing any action contrary to his or her conscience under any circumstance, that is, he or she will be under no obligation to provide any good or service that violates his or her conscience, not even indirectly to facilitate a patient’s access to it, e.g. by referring her to a non-objecting provider. They exemplify what Mark Wicclair (2011) calls “conscience absolutism.”

As a reaction, some reproductive rights advocates claimed that, in the context of health care, CO should not be allowed, since the right to legal termination of pregnancy is guaranteed by the law, and healthcare personnel working in public hospitals have a professional obligation to provide legal abortions when requested by women meeting the requirements.^6^ They also claimed that there are certain commitments made by people who want to devote themselves to medicine or work in the healthcare sector and that if their beliefs prevent them from fulfilling those commitments, then they should do something else; as physician Patricio Santillán-Doherty claimed, they should “object to their inclusion in the medical guild” (2014, p. 173). Many people have taken up what Wicclair calls the “incompatibility thesis,” which holds that “it is contrary to the professional obligations of physicians, nurses, and pharmacists to refuse to provide any legal good or service within the scope of their professional competence” (Wicclair, 2011, p. xi). In the general debate over CO, there are many incompatibilists. Julian Savulescu, for example, claims that

If people are not prepared to offer legally permitted, efficient, and beneficial care to a patient because it conflicts with their values, they should not be doctors. Doctors should not offer partial medical services or partially discharge their obligations to care for their patients. (Savulescu, 2006, p. 294)^7^

Savulescu thinks that physicians have no right to refuse medical assistance in cases of abortion – as well as other procedures. However, it may not be feasible to tell people who are already healthcare providers that they should do something else. And even people considering a career in medicine, and who want to follow their vocation, may object to certain procedures on grounds of conscience.^8^ Not allowing CO may discourage people who may be good physicians or nurses, but whose core values conflict with certain procedures, such as abortion. Practicing medicine should not be taken as an all-or-nothing parcel (see Cowley, 2016, p. 360). People who oppose recognizing CO in medical practice tell us that a healthcare professional who refuses to perform an abortion is acting in a way that jeopardizes women’s health and lives. The right to health care, they claim, and reproductive rights in particular, should not be subordinated to the religious or moral beliefs of healthcare personnel.

An additional reason that has been used for opposing the recognition of a right to CO in health care has to do with the fact that, in Mexico, there are not enough physicians to take care of the health needs of all the population. A study by the Joint Learning Initiative suggested that countries with fewer than 2.5 healthcare professionals per 1000 inhabitants (counting only physicians, nurses and midwives) failed to achieve an 80% coverage rate for deliveries by skilled birth attendants (Chen et al., 2004). In Mexico, there are 1.9 physicians per 1000 inhabitants (European countries have in average 3.06 [World Bank, 2017]). Most physicians are not specialized, and half of all of them are distributed in the five wealthiest states of the country; the other half in the remaining 27 states, where there are communities with no doctors at all. Furthermore, taking into account that most of the population in the country is Catholic, then it is very likely that, if the right to CO is recognized without restrictions throughout the country, there will not be enough physicians to perform the procedures associated with the termination of pregnancy as well as other procedures that some people find objectionable.

However, it seems to be inevitable that there will be cases of CO in medical practice and many of these cases are justified and respond to a right to freely hold and practice conscientious religious, philosophical, and moral convictions, on which the possibility of CO is based. Against incompatibilists, I shall argue that there is a right to CO: there are moral reasons that justify it and it is a right that may be invoked not only in medicine, but also in others areas. However, this right should not be seen as absolute, exempting objectors from performing some compensatory work or, in some cases, the very procedures they oppose. We should look for a compromise that accommodates the right to CO and women’s acknowledged sexual and reproductive rights when they conflict. This is supported by Mexico City’s General Health Law article cited above. However, the law should be more specific in the way it regulates CO.

In order to argue for this approach, we must look more closely at a number of issues: first, what are we referring to when we talk about CO and how should we understand it; second, what is its justification, particularly since it is not often recognized in legal codes. Once we answer these questions, we will be in a better position to analyze the issue that is being debated in Mexico as well as in other countries: how to regulate CO when it conflicts with other rights, particularly sexual and reproductive rights. It is not so much the recognition of CO that may obstruct women’s access to abortion as its lack of regulation.

## CO: justifying a right

CO arises when a person refuses to act according to a legal mandate or obligation, or an administrative order, on conscientious grounds, that is, on the basis of reasons for doing what this person believes is right (moral, religious or else), and which are opposed to such an obligation or order. CO reveals a conflict between an individual’s moral values and what he or she is being required to do.

CO is rarely recognized within the legislations of most countries, and sometimes it is regarded as a phenomenon to be tolerated rather than as a right. Thus, appealing to a right to CO is something that has to be justified in moral terms; there is a moral right to object in conscience. Moral rights are rights that are not necessarily recognized by a positive legal order, but which are valid claims, or sufficiently justified in moral terms so as to give rise to a legal right; as such, the enjoyment of the object of the right needs to be protected against possible threats and should be in some way promoted. These are not to be identified with the rights that arise from the norms of positive law, but, in any case, as Carlos S. Nino stated,

it is understood that the legal rights thus created constitute only a consecration, a recognition or a means of implementation of those rights that are logically independent of this legal reception. Respect for human rights is demanded even against legal systems that do not recognize them and *precisely because they do not recognize them*. (Nino, 1989, p. 15, his emphasis)

Thus, although a legal system may not recognize the right to CO, people may demand its recognition in terms of the moral reasons that lie behind it.^9^ These are also the bases for the justification of the right to freedom of conscience, which aims to protect the most intimate realm of personal convictions from considerations of social utility or from a government’s interference. The moral justification for recognizing a right to CO, in the various fields in which such recognition is called for (including health care), can be achieved in at least two ways, in terms of (1) respect for moral integrity or (2) respect for autonomy.^10^

The first line of moral justification is in terms of respect for moral integrity. Someone has moral integrity when she has a set of core moral values or principles and wants to act according to these values (Wicclair, 2011, p. 25). These are an important part of the identity of a person, part of the conception she has of herself. To require someone with moral integrity to act against the values and ethical principles that make up her identity would mean demanding that she dissociates from herself, from what constitutes her own identity. Thus, someone with moral integrity refrains from acting against such moral principles, and appealing to conscience can then be understood as an attempt to maintain that integrity. The law should find ways so that people are not led to act against their core principles, as a form of respect to the individuals and as a way to promote a moral virtue that societies should endorse.

The second line of defense is based on the idea of respect for individual autonomy. Autonomy is the ability each of us has to live our own lives according to the reasons and motives that we take as our own and that are not the product of external forces. Individual autonomy includes our capacity to provide for ourselves the moral values and principles with which we choose to guide our lives; in this sense, it is not only the ability to determine what moral principles or values we want to govern our lives, but which principles, reasons or motives are to rule our actions and our lives in practical terms. Neither the state nor other social institutions should ignore autonomy; on the contrary, this is a moral virtue that every society should promote. Respecting individual autonomy implies, above all, a pluralistic view that accepts that people have the right to act according to their own moral convictions, however diverse. In the words of Nino, the principle of autonomy prescribes that

since individual free choice regarding life plans and the adoption of ideals of human excellence are valuable, the state (and other individuals) should not interfere with that choice or adoption, limiting itself to designing institutions that facilitate the individual pursuit of these life plans and the satisfaction of the ideals of virtue that each holds and preventing interferences in the course of such pursuit. (Nino, 1989, pp. 204–5)

In any case, integrity and autonomy should only be limited in order to prevent harm to other individuals.

Both lines of moral justification can together support the appeal to the right of freedom of conscience, and of CO in particular.

However, a common reaction to viewing CO as a right is that, we are told, recognizing it as a right implies introducing as a legal concept the right to disobey the law, thus undermining the authority of the law and the general obligation to obey it. Recognizing a right to CO implies that those who invoke it would be authorized to disobey any established legal regulations. Anyone could end up invoking this right to disobey any legal rule that he thinks goes against his moral principles. In this sense, the refusal to recognize CO is due to fear of taking the first step on a “slippery slope,” where we may end up completely undermining the force of law. This fear is unjustified: recognizing a right to object to a specific legal norm does not imply that we will end up recognizing a right to disobey all or any norms. In those countries where a right to CO has been legally accepted in health care or in the army, people have not used it to argue for a right to disobey other laws, even less for a general right to disobey the law. There are justifications for invoking the right to objection in specific areas such as military service – which, by the way, is not legally recognized in Mexico – or health care, and we do not have to generalize these justifications to cover any norm or to think that we will find ourselves on a slippery slope. There are fallacious and non-fallacious uses of “slippery-slope” arguments, and this is a fallacious one.^11^

I want to emphasize that CO is an individual’s right: it is always the expression of a personal position, of the individual conscience of a person, which is intended to protect a private sphere from government or institutional intervention. Collective agents, such as hospitals, clinics and other institutions – even if they have a moral character and legal personality, and are capable of making legal claims in their own right – have no claim to moral conscience and cannot invoke the right to CO as individuals can. No public hospital can refuse to perform abortions, since the right to terminate a pregnancy is a right recognized by the state, and it would be contradictory for these institutions not to comply with the law. Private hospitals may be free to decide which procedures they offer to their patients, but if they offer gynecological and obstetrical procedures, and these sometimes result in abortions, as an institution they should not object to performing them. The right to object, in any case, would be a privilege of healthcare personnel. Institutions, on the other hand, could be sued if, for ethical or religious reasons, they prohibit the performance of procedures indicated on medical grounds.

## Conflict of rights: abortion and CO

So far I have talked about how to understand CO, and about its justification as a right. However, to understand CO as a right does not mean that it has to be seen as some kind of absolute right that overrides any mandatory legal rule or another person’s rights, or that individuals who invoke it do not have any obligation to provide a good or service that violates their conscience, nor even to indirectly facilitate patients’ access to it by referring them to someone else. To recognize a legal right to objection implies that the state is obliged to respect the healthcare personnel’s religious or ethical convictions, but at the same time, since the state also acknowledges the rights of women to safely terminate their pregnancies, this entails that it is obliged to guarantee the conditions under which that right can be fulfilled. In many cases the two rights may coexist without conflicts, since there may be non-objectors who take care of those women who require abortion procedures, but in other cases this may not be feasible, and then the right to CO would have to be restricted. There are moral as well as legal reasons for restricting the exercise of CO and reaching a reasonable balance between the conscience of healthcare providers and women’s healthcare needs and interests when they need or want to have an abortion. A compromise should be reached between these two rights.

Among the reasons to restrict the right to CO, we find some of the core professional obligations of healthcare personnel: the obligation to respect patient dignity and refrain from invidious discrimination, CO has also to be compatible with the obligation to promote patient health and well-being, and with the obligation to respect the patient’s autonomy (Wicclair, 2011, p. 88). The professional codes of some of the most important Mexican medical associations, such as the National Academy of Medicine, the Mexican Academy of Surgeons or the National College of Nurses, recognize these obligations.

Sometimes, a refusal to provide a service may be based on the belief that certain classes of patients are not entitled to it. This kind of refusal may be based on invidious discrimination, and in this case it is contrary to the professional obligations of these healthcare providers and is ethically unacceptable, since it does not respect the dignity of patients and their right to equal treatment. Discrimination is the treatment or consideration of a person or a group of persons based on the category (race, color, language, political opinion, religion, age, sexual orientation, national origin, etc.) to which such individuals are perceived as belonging rather than on individual attributes. If an objector were to refuse to perform an abortion on the basis of discriminatory beliefs, say, just because the woman asking for the service is a sex worker or because she belongs to an indigenous community, this would be ethically unacceptable. However, most refusals to perform abortions are not based on discriminatory beliefs: conscientious objectors do not generally refuse to perform abortions to women just because they are women or because they are perceived to belong to a certain category of people, but because they think that an abortion involves the killing of a human being. It may be argued, however, that it is discriminatory for a state, an institution or an individual to refuse to provide a certain health service that is exclusive to women, such as abortions. Restrictive abortion laws, it is alleged, infringe the rights to privacy, equality, and autonomy of women, while legislation recognizes these to men, so there are grounds for thinking that these laws are discriminatory.^12^

Another argument against absolutism of conscience is that the right to CO may also be limited or conditioned by the patients’ rights. Mexico’s Supreme Court justice José Ramón Cossío, in his opinion in favor of Mexico City’s abortion law, argued that the Constitution does not recognize absolute rights. The Constitution requires reconciling a set of values that are not necessarily compatible, that is, it must balance opposing values that may conflict, such as the fetus’s alleged right to life and women’s right to decide over their own bodies and maternity (Cossío, 2008). If the right to life, as he argued, is not an absolute right, since there are cases in which the woman’s right weighs more, neither is the right to CO. The latter has to be balanced against other rights, such as the rights of patients. Since the right to CO is a limited right, conditioned by the context, “conscience absolutism” should be rejected. There are circumstances in which this right has to be conditioned and the opposing right should be prioritized.

The right to CO in health care gets into conflict with women’s legally recognized right to terminate their pregnancy, which is precisely the procedure that objectors refuse to perform. There are cases where treatment is necessary to save the life of a pregnant woman or to prevent serious permanent injuries to her health, and where the objector is the only person available to treat her. In 2006, before elective abortion was decriminalized in Mexico City, the DoH had already stated that

Health professionals may refrain from participating in the practice of legal termination of pregnancy [in reference to those indications legal at the time] on grounds of conscience, except in cases in which the life of the pregnant woman is at imminent risk. (DoH, 2006)

What would be the justification in these cases? Just that, balancing rights, the harm to the health, physical integrity and future well-being of women weigh more than the harm to the conscience of the objector. The FIGO Committee for the Ethical Aspects of Human Reproduction and Women’s Health concurs: “The primary conscientious duty of OB/GYNs is at all times to treat, or provide benefit and prevent harm to, the patients for whose care they are responsible. Any conscientious objection to treating a patient is secondary to this primary duty” (FIGO, 2012). Even from the perspective of the Catholic doctrine of double effect these cases may be justified, since the pursuit of one’s otherwise legitimate ends (e.g. trying to save the life of a woman in cases of ectopic pregnancies) may cause some foreseeable effect that, in other circumstances, one would be obliged to avoid (terminating a pregnancy).

The law must establish a system that allows, at the same time, the exercise of the right to CO and the fulfillment of the right to a legal termination of pregnancy, that is, CO should be regulated so that it is not used to obstruct women’s access to safe abortion. Some of its very basic aspects have to be regulated in order to protect possible obstructions of women’s reproductive rights. When these two rights clash, they have to be balanced, according to the context, by the hospital ethics committee, but also with the aid of Mexico’s National Bioethics Commission (Conbioética). This Commission, which coordinates hospital ethics committees, should deal with cases of CO, and these conflicts should be resolved according to guidelines and standards. The Commission, along with ethics committees, is in charge of ensuring that the rights of those accessing health services are guaranteed. The guidelines of the hospital ethics committees (Conbioética, 2012), derived from the federal General Health Law, establish that these committees are the bodies in charge of internally regulating moral situations that may affect the patients’ rights. Therefore, when someone declares himself an objector, these committees should evaluate this declaration to ensure that the necessary measures to guarantee health care are covered.

In cases where there is a possibility that CO may affect the rights of patients, the state has to ensure that there are enough non-objecting physicians and healthcare personnel in public hospitals performing abortions on women requiring this procedure in the exercise of their reproductive rights.^13^ The state must guarantee the right to CO, and at the same time access to, and quality of, the service. Recognition of the right to termination of pregnancy does not simply mean that the state is not penalizing women who decide, for whatever reason, to interrupt their pregnancies, but it also implies that the state ought to make sure that there will be enough personnel to provide the service (this may sometimes be a goal hard to achieve; as has already been mentioned, in Mexico there are not enough physicians to go round, which implies that the state must invest more in satisfying the need for more human resources in health care). It should also provide the safety conditions under which women carry out their abortions. This is true in Mexico City, where the local government committed itself from the outset to implement, first in public hospitals, then in specialized health centers, an abortion program where women receive counseling, as well as medicines and, whenever needed, surgical procedures, to perform abortions at no cost for Mexico City residents (there is a sliding scale for out-of-state residents, averaging US$85).

## The need to regulate

There is a need to regulate CO in medicine, since leaving it unregulated and at the discretion of each individual or each health center produces legal uncertainty and may obstruct the rights of others, particularly women’s sexual and reproductive rights. This is why conscientious objectors must declare their objection when they start working at an institution, or when a certain program is implemented, so that the institution can plan, ahead of time, how to distribute its personnel and thus be in a position to provide services. There must be a registry of conscientious objectors run by the DoH in order to ensure that there are enough non-objectors at each hospital or clinic. In order to regulate, there are some questions that need to be addressed; basically, the problems revolve around some fundamental questions: *who* can object, *how* they can object, and *what* they can object to.

- *Who can object?* The right to CO, as an instantiation of the right to freedom of conscience, is a human right; so in principle it is a right that any person can exercise if she is in a position where she has to act according to a legal mandate or obligation, and where this goes against what she believes is morally right. In that sense it is not a monopoly of OB/GYNs and general surgeons: in principle anyone who directly or indirectly participates in an abortion procedure ought to be able to object in conscience. There has to be a good reason if we are going to deny or restrict the right to CO to someone who in any way participates in the procedure: midwives, anesthesiologists, social workers, nurses, pharmacists, but also the administrative staff of the hospital. As already mentioned, Mexico City’s General Health Law recognizes the right to CO only to physicians who could be in charge of performing the abortion. Along these lines, the Procedures Manual for Legal Terminations of Pregnancy states “The medical, nursing and paramedical personnel who assist the obstetrician-gynecologist or the general surgeon in the legal interruption of the pregnancy, under no circumstance, may invoke conscientious objection” (DoH, 2008). If the law recognizes this right to physicians, but not to the rest of the personnel involved, it is because their role is auxiliary or secondary, since they just facilitate the procedure conducted by the physicians. Their level of involvement is indirect or remote, so this severs their responsibility (Dickens & Cook, 2000). Anyhow, even if the law were to allow the right to CO to the rest of the personnel involved directly or indirectly in the procedure, and a nurse or a member of the administrative staff of the institution were to object in conscience, they would have to do so in advance, so as to enable the institution to plan ahead of time and so that this request might go through its ethics committee and, in turn, to the National Bioethics Commission.

Hospitals’ ethics committees should have a more active role in assessing cases of CO. Just as in the US army – where objectors have to satisfy a tribunal regarding the sincerity of their objection to war, and to confirm that it is based on ethical or religious beliefs about what it is right or wrong – healthcare professionals should justify their objection to the ethics committee (see Clarke, 2017). Since the word of the person invoking the right to objection is often the only direct evidence of his moral convictions, the opportunities for abuse may be high. For example, some cases have been reported in which physicians of public hospitals run by Mexico City’s DoH have invoked CO in order not to perform abortions, when the real motivation for their actions was to avoid increasing an already heavy workload; in some other cases, they were objectors in their public service, but not in their private practice (Contreras et al., 2011 and Díaz-Olavarrieta et al., 2012).

It is particularly crucial that the administrative staff in charge of the program be non-objectors, since otherwise the hospital may not have the necessary supplies to carry out the procedures the objectors refuse to cater for. Nor should members of the DoH in any way in charge of the management of programs or the administration of hospitals be objectors if their objection could jeopardize the proper management of the services and, therefore, affect the rights of women who want to interrupt their pregnancies. Therefore, the DoH has to make sure that these positions be occupied by non-objectors.

Sometimes, people declare themselves to be COs either out of fear of social stigma or because they are unaware of their professional obligations. In order to deal with these cases, the DoH may, for instance, implement values clarification workshops for healthcare personnel, such as those of IPAS (Gómez Ponce de León & Turner, 2009), which try to eliminate obstructionist behaviors based on erroneous information, negative attitudes and stigmatization, or may disseminate the evidence that making legal abortion more available does not increase abortion rates, but on the contrary, reduces maternal mortality and morbidity (Faúndes, Duarte, & Osis, 2013).

- *How is conscientious objection to be declared?* CO must be explicitly stated so that the health institution can plan the provision of its services in advance. These institutions have organizational needs and have to anticipate the best conditions under which they can ensure the fulfillment of their obligations and the provision of services to those entitled to them. For the declaration of CO, public institutions would have to establish the conditions, requirements and deadlines for being able to exercise this right. For example, a declaration of objection could be made through a written statement specifying which procedures someone objects to so that the information can be included in a centralized registry, available to the health center or hospital and the DoH, and protected so as to ensure the objector’s right to privacy.^14^ (The state has to guarantee the conditions for exercising that right, so it must ensure that reasonable conscientious objectors are not punished, coerced, held liable, discriminated against or forced in any way when refusing to perform, or to assist in, an abortion or any other specified procedure. In the same way, nobody should be discriminated against for performing legal abortions.)
- *What exactly does a person object to?* The statement of CO must tell exactly what it is that is objected to. Although the right to CO has been demanded basically for terminations of pregnancy, there are many other services in addition to abortion that can be subject to CO: prenatal genetic screening, assisted reproduction techniques, such as IVF or surrogacy, among some others. Since some of these are relatively frequent procedures (and may be more so in the near future), it is necessary that the objector explicitly state which procedures she refuses to perform. Even in the case of abortion, the objector may refuse to perform some types of abortions, but not others; for example, she might agree to perform abortions when the embryo presents a serious congenital or genetic anomaly, when pregnancy is the result of rape or when the woman’s life is at risk, but refuse to perform abortions when these are elective. As a matter of fact, people have different moral beliefs about different causes of abortion. In a 2004 survey, 71% of Mexican physicians said that they would agree to perform an abortion for a woman in case of rape, 85% if the woman’s life is at risk, and 70% in cases of severe fetal malformation (Population Council, 2004). People perceive relevant moral differences regarding different reasons for performing an abortion. As already mentioned, most state laws allow for these three causes, while penalizing elective abortion.

However, many may refuse not only to provide services that they find objectionable, but also to refer patients to other providers or even to inform them of contraceptive and pregnancy options counseling. As Schuklenk (2015, p. ii) rightly observes:

If I object to abortion because I believe that abortion is akin to murder, as Christian objectors happen to believe, surely my moral responsibility is barely smaller if I knowingly pass a pregnant woman looking for an abortion on to a colleague who will commit the act rather than if I do it myself.

It is hard to force people to facilitate situations they take to be morally wrong. However, if the objector refuses to provide the service to a woman seeking an abortion, and does not even agree to refer her to a non-objecting physician, then he is failing in his professional obligation to respect her autonomy. Additionally, if the patient’s physical or mental well-being in any way depends on this kind of information, then the objector should comply with his professional commitment to assist her, particularly if the patient is in a state of urgency or great need. Failure to refer a patient to a non-objecting physician may risk exposing her to significant harm. Chervenak and McCullough (2008) see this professional duty to protect a patient as the basis of an obligation to refer. However, should the objecting healthcare provider, acting against his professional commitments, not wish to refer her to a non-objecting physician, the DoH, through the hospital or health center, must provide women with information about non-objecting doctors. Yet, finding the right information may be difficult for some women, and this is why sometimes the law requires doctors to provide women with a list of alternative non-objecting doctors or to refer to one in particular.

However, as Clarke (2017) has pointed out, physicians who conscientiously refuse to perform abortions at an institution in which other doctors do perform them make an indirect contribution to abortions taking place. By working at the same institution, as often happens in Mexico City, they absorb part of the alternative workload from non-objecting colleagues who thus have more time to perform abortions; in so doing they share the responsibility for abortions taking place. The upshot of this, Clarke argues, is that objectors working at institutions where abortions are performed should be obliged (morally and legally) to refer to others for abortions. Those who recognize their complicity and strongly object should be helped to find work somewhere else, and be exempted from referral – the same would go for personnel who have an auxiliary role in abortions, such as nurses or paramedical personnel.

Finally, it is true that the phenomenon of CO in Mexico City occurs basically in public hospitals where health personnel are under the obligation to provide a service that the city recognizes as a right and is committed to provide. Nevertheless, this phenomenon can also occur in private hospitals where doctors are under the obligation to provide abortion services if that is the policy of the hospital. This phenomenon tends to occur to a much lesser extent in private hospitals, since many either simply do not have the policy of offering abortion services, or else it is in their interest to have enough non-objecting personnel to provide the service. It might be claimed that if it is possible for a woman to go to a private clinic to have an abortion, then CO would not have to be an obstruction for the woman to have an abortion. After all, someone might say, there are dozens of private clinics that currently provide abortion services in Mexico City.^15^ Private hospitals and clinics registered with the DoH guarantee abortion services in optimal conditions of hygiene and safety. Then, what is the fuss about CO? First, because Mexico City’s authorities have to ensure a compromise between women’s right to reproductive health care, on the one hand, and the exercise of CO, on the other – especially in their own clinics. Second, because the DoH is committed to providing free or low-cost health services for women who cannot afford the cost of an abortion in a private clinic (ranging from US$50 to 600 [IPAS, 2011]). Otherwise, it is likely that, as has happened in the past and also happens in the rest of Mexico and in most of Latin America, only women who can afford to have an abortion in a private hospital or clinic have the ability to exercise this “right,” whereas poor women do not. They are the ones who have traditionally been denied the right to choose.

## Notes

1 There was a previous reform in 2003 in which the city’s General Health Law was modified to admit physicians’ CO (GIRE, 2008). Both the 2003 and the 2009 reforms considered only physicians, and not nurses or other health care personnel, although the last sentence refers to “non-conscientious personnel in the field,” somehow implying the latter. The federal norm regulating services provided to women in cases of sexual violence (NOM-046-SSA2-2005) only states that “the CO of the medical and nursing staff in charge of the procedure must be respected,” but it does not say much more about regulations.

2 During 2008, some 21,600 private-sector induced abortions were performed in Mexico City, involving at least one in five of the more than 3,000 facilities identified (Schiavon et al., 2010). In comparison, approximately 13,000 women had an abortion in public health facilities during the same year. By 2011, the number of abortions in private clinics had quadrupled (IPAS, 2011), and public hospitals perform, in average, some 17,000 abortions every year. However, there is no information about cases of CO in private hospitals or clinics. Thanks are due to Martha Pérez Martínez, of the Department of Reproductive Health of the DoH, for this information. Up-to-date information about abortion in Mexico City can also be found at http://ile.salud.cdmx.gob.mx/estadisticas-interrupcion-legal-embarazo-df/.

3 However, in some states in Mexico where abortions performed under the three grounds cited are legal, there are no reported cases of legal abortions being provided; even when in those states there are estimates of thousands of unsafe abortions. This is mostly due to the obstructionist behavior of the health care personnel, district prosecutors and judges. See Lamas (2017, p. 171).

4 For a brief history of the path that led to the decriminalization of abortion in Mexico City, see Madrazo (2009), Ortiz-Millán (2016) and Lamas (2017).

5 This is something that is also going on in other countries in Latin America, such as Colombia, Chile and Peru, where progress in reproductive matters has been achieved in recent years. See Casas (2009).

6 In April 2007, Mexico City’s head of government at that time, Marcelo Ebrard, said that health care personnel should abide by the provisions of abortion on demand, under penalty of being dismissed, and that they could not appeal to conscientious objection, since it did not apply, given that health care personnel were public servants “forced to abide by the disposition included by law,” since “abortion is a right” (Cedillo Cano, 2007). However, in the end the possibility of objecting on grounds of conscience became legally accepted.

7 See also Schuklenk (2015) for a similar position. Blustein and Fleischman (1995) also argue that since maternal-fetal medicine sometimes results in abortions, pro-life OB/GYNs may have a hard time maintaining their integrity, so they should not enter this medical field.

8 Analogously, students in the natural sciences may object to participating in certain practices, such as the vivisection or the dissection of animals (as already recognized in a number of laws), but accept all other practices involved in their studies. The right to CO in the use of animals in education was recognized in Mexico City’s 2014 Animal Protection Law (art. 46).

9 But also as deriving from rights explicitly recognized in international law, such as the Universal Declaration of Human Rights (art. 18) and the International Covenant on Civil and Political Rights (art. 18, par. 3). In Mexico, the Constitution protects the right to freedom of ethical convictions, conscience and religion (art. 24).

10 See Wicclair (2000, pp. 205–227, and 2011, pp. 25–31). Wicclair discusses the problems that the justification of CO may have in these terms, as well as other possible justifications. Lewis-Newby, Wicclair and their colleagues (2015) also discuss improving the quality of medical care (through allowing diversity) and identifying changes needed to professional norms by way of accommodating CO in medicine. However, these arguments do not directly justify a right to CO.

11 On the other hand, as Ronald Dworkin (1978, chapter 8) has pointed out, the duty to obey the law and the state is not an absolute duty, since any state, even though it has a constitution and a publicly accepted legislation, can establish specific laws that some citizens may consider unfair and that may conflict with their moral or religious convictions. While citizens have legal duties, they may also have religious or moral duties that conflict with them, and legal codes should recognize the possibility of such cases.

12 In 1999, the Committee on the Elimination of Discrimination against Women (CEDAW) general recommendation no. 24 (paragraph 11) categorized as discriminatory the prohibition of abortion: “It is discriminatory for a State Party [to the Convention] to refuse to legally provide for the performance of certain reproductive health services for women.” Then it refers to CO: “For instance, if health service providers refuse to perform such services based on conscientious objection, measures should be introduced to ensure that women are referred to alternative health providers.” On the issue of whether restrictive abortion laws are discriminatory, see Butler (1991).

13 The Mexico City 2003 reform maintained that hospitals must have non-objecting staff to provide all legal abortions. It was not enough that women could be referred to another public hospital but that, in fact, the same exact hospital must have non-objecting staff. This was before the 2007 abortion reform and the ILE program, and only applied to those indications legal at the time. See DoH (2006).

14 A proposed model of a declaration of conscientious objection in medicine can be found in Casado and Corcoy (2007).

15 Although private clinics are not required to make their statistical records available for abortions, as is the case for public hospitals and health centers run by the city’s DoH, it is estimated that three-quarters of the abortions performed in Mexico City take place in private clinics.
